# Which scoring system is better in association with exercise capacity and health status in noncystic fibrosis bronchiectasis patients?

**DOI:** 10.3906/sag-2005-18

**Published:** 2021-04-30

**Authors:** Yelda VAROL, Hülya Doğan ŞAHİN, Nimet AKSEL, Ali Kadri ÇIRAK

**Affiliations:** 1 Department of Chest Diseases, University of Health Sciences, Dr. Suat Seren Chest Diseases and Surgery Education and Training Hospital, İzmir Turkey

**Keywords:** Noncystic fibrosis bronchiectasis, FACED score, BSI score, 6-min walking distance, health status

## Abstract

**Background/aim:**

Two different scoring systems were developed to determine the severity of bronchiectasis: FACED scoring and the bronchiectasis severity index (BSI). In this study, we aim to compare these 2 scoring systems according to the 6-min walking distance test and a disease-specific health status questionnaire in patients with noncystic fibrosis bronchiectasis (NCFB).

**Materials and methods:**

Smoking history, emergency and hospital admissions, and body mass index were obtained from NCFB patients admitted to our hospitals’ pulmonary rehabilitation unit between 2013 and 2018. Detailed pulmonary function tests were performed for all participants. Dyspnea perceptions were determined according to the mMRC dyspnea scale. The 6-min walking test was used to determine exercise capacity. The Saint George respiratory questionnaire (SGRQ) was applied to determine health status. Both FACED and BSI scores were calculated for all participants.

**Results:**

There were a total of 183 participants, 153 of whom were men. A significant and strong correlation was found between FACED and BSI scores. As the severity of bronchiectasis increased, walking distance was significantly decreased and health status was significantly worse in both FACED and BSI scoring. A statistically significant but weak negative correlation was found between FACED score and walking distance. There was a significant negative correlation between BSI and walking distance, a stronger negative correlation than with FACED. Similarly, there was a significant negative correlation between health status and both FACED and BSI, but this correlation was stronger in the BSI score.

**Conclusion:**

Although both FACED and BSI scores were negatively correlated with walking distance and health status in patients with NCFB, BSI was more strongly associated.

## 1. Introduction

Bronchiectasis, which is now termed muco-obstructive lung disease, is actually an orphan disease [1]. Bronchiectasis is a clinical-radiological diagnosis characterized by irreversible airway dilatation with chronic inflammation, which has the clinical features of cough, sputum production, and episodic exacerbations [2]. 

Bronchiectasis is classified into 2 categories, noncystic fibrosis bronchiectasis (NCFB) and cystic fibrosis bronchiectasis (CFB). NCFB has gained attention with increasing awareness. With the increased use of high-resolution CT scan, more patients have received a diagnosis of bronchiectasis. The severity and the prognosis of NCFB cannot be determined by only one parameter. Therefore, there are several validated scores in determining the severity and prognosis of this disease. Two of these are the FACED score and the bronchiectasis severity index (BSI) [3,4]. The FACED score is a 5-point score that predicts probability of all-cause mortality. The FACED score consists of forced expiratory volume in 1 sec (FEV1) % predicted, age, chronic colonization by *Pseudomonas aeruginosa* , extension of the disease by radiological assessment, and dyspnea [3]. However, BSI score is a 7-point score that determines risk for future mortality and hospitalizations for NCFB patients [4]. In addition to the criteria of the FACED score, the number of emergency visits and number of hospitalizations are also calculated in the BSI score. 

Unfortunately, there are no studies comparing the 2 scores in association with exercise capacity and health status in patients with NCFB. Therefore, in the present study we aimed to compare the results of the assessment of 6-min walking distance and health status (SGRQ) parameters in the same patients with NCFB assessed with both the FACED score and the BSI.

## 2. Materials and methods

We conducted a retrospective database study in our Chest Diseases and Surgery Education and Training Hospital between 2013 and 2018 to compare the results of the assessment of 6-min walking distance and health status parameters in patients with NCFB assessed with both the FACED score and the BSI. The study was approved by the local institutional review board. Patients included in the study completed an informed written consent form. 

### 2.1. Subject selection

All patients with NCFB were referred to our Pulmonary Rehabilitation Unit for the PR program. Of the 218 NCFB patients, 183 were eligible to participate the study. The inclusion criteria were having bronchiectasis on high resolution computerized tomography (HRCT) of the lungs with or without airway obstruction. The exclusion criteria were having an interstitial lung disease pattern (to exclude traction bronchiectasis) on HRCT, having concurrent lung cancer, or having a history of having had a lobectomy or pneumonectomy for lung cancer. 

### 2.2. Measurements

Age, sex, and body mass index (BMI) as demographics and clinical history (smoking history, colonization with *P. aeruginosa* , radiologic severity according to HRCT, emergency admission, and hospitalization in the last year) were recorded.

### 2.3. Respiratory functions

Body plethysmography (ZAN 500, nSpire Health GmbH, Oberthulba, Germany) and carbon monoxide diffusion capacity (ZAN 300) are routinely measured for all patients with NCFB who are admitted to our hospital PR unit [5]. We recorded the % predicted values of FEV1, FVC, FEV1/FVC ratio, and carbon monoxide diffusion capacity (TLCO). 

### 2.4. Dyspnea assessment

We used the modified Medical Research Council (mMRC) dyspnea scale, which consists of 5 items ranging between 0 and 4, to determine the severity of the patients’ shortness of breath. The score 0 represents the best level, while the score 4 indicates the poorest [6].

### 2.5. Exercise capacity

We recorded the walking distance in the 6-min walking test (6mWD) performed according to the American Thoracic Society (ATS) guidelines [7].

### 2.6. Health status

We used the St. George’s respiratory questionnaire (SGRQ) to determine disease-specific health status [8]. The participants’ psychological status was determined by the hospital’s anxiety depression scale [9]. All of these questionnaires are routinely given to all of our pulmonary rehabilitation candidates.

### 2.7. Bronchiectasis indexes

Both FACED and BSI scores were calculated for all participants, and participants were divided into 3 groups as mild (0–2 for FACED, 0–4 for BSI) moderate (3–5 for FACED, 5–8 for BSI), and severe (6–7 for FACED, >9 for BSI). All data, especially walking distance and health status data, were compared between these 2 scoring systems. A bronchiectasis exacerbation was defined as a patient with bronchiectasis with deterioration for at least 48 h in ≥3 of the following symptoms: cough, sputum volume and/or consistency, sputum purulence, breathlessness and/or exercise intolerance, fatigue and/or malaise, hemoptysis [10]. 

### 2.8. Statistical analysis

The Statistical Package for the Social Sciences (IBM Corp., Armonk, NY, USA) v. 22 was used for data analysis. Before the statistical analysis, the parameters were tested for normal distribution with Kolmogorov–Smirnov and Shapiro–Wilk tests. Results are presented as mean ± standard deviation, median (minimum-maximum), number (n), or percentage (%) according to the statistical method. One-way ANOVA test was used for continuous variables when comparing clinical data of patients between the BSI and FACED groups. As the number of patients differed between the groups and homogeneity of variance was provided, Hochberg’s test was used for posthoc analysis. Hochberg’s test allows for clearly unequal sample sizes. The relationship between the BSI and FACED scores of the patients and the relationship between the 6-min walking distance measurement values and SGRQ health status scores of the patients were demonstrated by simple linear regression analysis and Pearson’s correlation test. P values <0.05 were considered statistically significant.

## 3. Results

There were 183 participants, 153 of whom were men. The mean age of the study population was 63.1 (±10.4). The mean FEV1 % was 33.5 ± 17.6 and the mean FEV1/FVC ratio was 56.8 ± 14.1. There were 64 (34.9%) patients who had the diagnosis of bronchiectasis with COPD, 7 (3.8%) with a diagnosis of bronchiectasis with asthma; the remaining were classified as isolated bronchiectasis before referral. Nearly half of the patients (46.8%) had at least one comorbidity and 8 (4.3%) patients had sequel tuberculosis. The mean FACED score of the population was 3.4 ± 1.5 while the mean BSI score was 8.9 ± 4.6. The distribution of severity according to FACED and BSI scores is shown in Table 1. The mean 6mWD was 331.8 ± 114.6 m. The other demographic characteristics and the QoL scores are shown in Table 1. According to FACED bronchiectasis classification, patients in the severe group were older and had higher SGRQ scores and a lower 6mWD compared to those of the patients in the mild and moderate groups; all differences were significant (for all, P < 0.01) (Table 2). HAD scores were not significantly different between FACED severity groups (P = 0.97 for anxiety and P = 0.91 for depression) (Table 2). According to the BSI index, patients in the mild group were younger, had higher 6mWD, and had lower SGRQ scores; all differences were significant (P < 0.01 for all) (Table 3). A statistically significant but weak negative correlation was found between FACED score and distance values; a statistically significant negative correlation was found between BSI and distance values, with a stronger correlation than FACED scores (Table 4). A 1-point increase in FACED score corresponds to a 28.4-m decrease in distance measurement; a 1-point increase in BSI score corresponds to a 13.2-m decrease in distance measurement. 

**Table 1:** Demographic data of the participants.

Age (years) (mean ± sd)	63.1 ± 10.4
BMI (kg/m2) (mean ± sd)	25.5 ± 5.4
Sex (n, %)MaleFemale	153 (83.6%)30 (16.4%)
mMRC (mean ± sd)	3.4 ± 1.2
Smoking history (pack-years) (mean ± sd)	55.0 ± 39.2
ER admissions (median) (min-max)	2.0 (0.0–20.0)
Hospitalizations (median) (min-max)	1.0 (0–10.0)
FEV1/FVC (mean ± sd)	56.8 ± 14.1
TLCO (mean ± sd)	35.4 ± 20.5
PaO2 (mean ± sd)	71.5 ± 13.0
PaCO2 (mean ± sd)	41.8 ± 6.2
SaO2 (mean ± sd)	93.5 ± 4.9
pH (mean ± sd)	7.4 ± 0.0
FACED score (mean ± sd)	3.4 ± 1.5
FACED scoring system (n, %)MildModerateSevere	46 (25.1%)114 (62.3%)23 (12.6%)
BSI scoring system (mean ± sd)	8.9 ± 4.6
BSİ scoring system (n, %)Mild ModerateSevere	38 (20.8%)57 (31.1%)88 (48.1%)
6mWD meters (mean ± sd)	331.8 ± 114.6
SGRQ symptom (mean ± sd)	57.8 ± 21.0
SGRQ activity (mean ± sd)	70.1 ± 20.3
SGRQ impact (mean ± sd)	53.5 ± 21.6
SGRQ total (mean ± sd)	59.2 ± 19.1

BMI: body mass index, mMRC: modified Medical Research Council, ER: emergency department visit, FEV1: forced expiratory volume in one second, FVC: forced vital capacity, TLCO: CO diffusion capacity, PaO2: partial pressure of oxygen in arterial blood gas analysis, PaCO2: partial pressure of carbon dioxide in arterial blood gas analysis, SaO2: saturation of oxygen in arterial blood gas analysis, 6mWD: six-min walk distance, SGRQ: Saint George respiratory questionnaire.

**Table 2:** Comparison of clinical characteristics and health status of patients according to FACED bronchiectasis classification.

	Mild n = 46	Moderate n = 114	Severe n = 23	P value	P* value	P** value	P*** value
Age	59.1 ± 8.8	62.8 ± 10.6	73.0 ± 5.0	<0.001	0.044	<0.001	<0.001
FACED score	1.4 ± 0.6	3.6 ± 0.6	6.0 ± 0.2	<0.001	<0.001	<0.001	<0.001
BMI	27.1 ± 4.2	25.4 ± 5.8	23.6 ± 4.7	0.051	0.285	0.055	0.386
6 mWD (meters)	395.5 ± 84.5	319.2 ± 120.4	266.3 ± 76.3	<0.001	0.045	<0.001	<0.001
SGRQ symptom	47.9 ± 18.1	59.8 ± 21.1	67.4 ± 19.5	<0.001	0.033	<0.001	<0.001
SGRQ activity	57.8 ± 17.7	72.9 ± 20.2	80.9 ± 14.7	<0.001	0.027	<0.001	<0.001
SGRQ impact	46.8 ± 18.7	53.7 ± 22.2	65.6 ± 19.4	0.003	0.038	0.002	0.041
SGRQ total	50.3 ± 16.4	60.5 ± 19.1	70.6 ± 16.6	<0.001	0.005	<0.001	0.043

* Mild vs. moderate, ** mild vs. severe, ***moderate vs. severe, one-way Anova test, Hochberg’s GT2 test for posthoc multiple comparison.

**Table 3:** Comparison of clinical characteristics and health status of patients according to BSI Bronchiectasis classification.

	Mild n = 38	Moderate n = 57	Severe n = 88	P value	P* value	P** value	P*** value
Age	57.5 ± 10.2	63.5 ± 9.4	65.3 ± 10.3	<0.001	0.004	<0.001	0.049
BMI	26. 5± 3.9	25.6 ± 5.3	25.0 ± 6.0	0.365	0.710	0.336	0.797
BSI score	3.2 ± 0.7	6.5 ± 1.2	13.0 ± 2.9	<0.001	<0.001	<0.001	<0.001
6mWD (meters)	409.3 ± 75.8	363.8 ± 98.6	277.5 ± 111.8	<0.001	0.043	<0.001	<0.001
SGRQ symptom	46.2 ± 19.3	54.5 ± 21.3	64.9 ± 18.9	<0.001	0.029	<0.001	0.006
SGRQ activity	57.7 ± 19.7	65.1 ± 21.3	78.7 ± 15.8	<0.001	0.031	<0.001	<0.001
SGRQ impact	44.5 ± 20.5	47.9 ± 21.7	60.9 ± 19.6	<0.001	0.045	<0.001	0.001
SGRQ total	48.7 ± 18.3	54.3 ± 19.1	66.9 ± 16.1	<0.001	0.037	<0.001	<0.001

* Mild vs. moderate, ** mild vs. severe, *** moderate vs. severe, one-way Anova test, Hochberg’s GT2 test for posthoc multiple comparison.BSI: bronchiectasis severity index.

**Table 4:** Correlation of the two bronchiectasis scoring systems with 6-min walking distance and health status scores: simple linear regression analysis.

FACED scoring system								BSI scoring system						
	r	B	95.0% CI	P	R2	F	P*	r	B	95.0 %CI	P	R2	F	P*
Walking distance(meters)	–0.373	–28.41	(–38.80)–(–18.04)	0.000	0.139	29.19	0.000	–0.533	–13.23	(–16.31)–(10.15)	0.000	0.284	71.68	0.000
SGRQ symptom	0.274	3.84	1.87– 5.81	0.000	0.075	14.73	0.000	0.416	1.90	1.29– 2.50	0.000	0.173	37.97	0.000
SGRQ activity	0.388	5.26	3.43– 7.09	0.000	0.151	32.13	0.000	0.477	2.10	1.53– 2.67	0.000	0.227	53.19	0.000
SGRQ impact	0.305	4.39	2.41– 6.45	0.000	0.093	19.59	0.000	0.407	1.91	1.28– 2.54	0.000	0.166	35.93	0.000
SGRQ total	0.360	4.56	2.83– 6.30	0.000	0.129	26.90	0.000	0.475	1.96	1.43– 2.49	0.000	0.225	52.68	0.000

Simple linear regression analysis.

There was a statistically significant but weak correlation between FACED score and SGRQ total, SGRQ symptoms, activity, and impact scores, and there was a statistically significant but weak negative correlation between FACED score and walking distance. There was also a statistically significant correlation between BSI and SGRQ total SGRQ symptoms, activity, and impact scores, and there was a statistically significant but weak negative correlation between BSI score and walking distance. The correlation between BSI score and SGRQ total score was stronger than the correlation between FACED score and SGRQ total score. A 1-point increase in FACED score corresponds to a 4.56 points increase in SGRG total score; a 1-point increase in the BSI score corresponds to 1.96 points increase in SGRQ total score (Table 4). There was a statistically significant and strong correlation between FACED and BSI scores (P < 0.001, r = 0.639). The increase in FACED accounted for about 41% of the increase in BSI (R2 = 0.409). When we compared the 6mWD among the mild, moderate, and severe FACED subgroups, there was no statistical significance between the subgroups (Table 5). However, in moderate and severe BSI subgroups, the 6mWD was significantly lower than that of the mild BSI subgroup (P = 0.034, P = 0.003) (Table 6).

**Table 5:** The correlation of 6-min walk distance and QoL scores in between FACED scoring system subgroups.

	Mild		Moderate		Severe	
	r	P	r	P	r	P
6mWD	–0.243	0.104	–0.117	0.214	–0.068	0.759
SGRQ total score	0.065	0.669	0.226	0.016	0.005	0.981

**Table 6:** The correlation of 6-min walk distance and QoL scores in between BSI scoring system subgroups.

	Mild		Moderate		Severe	
	r	P	r	P	r	P
6mWD	0.060	0.719	–0.281	0.034	–0.311	0.003
SGRQ total score	0.138	0.409	0.237	0.076	0.371	0.000

## 4. Discussion

In our cohort, 183 patients with NCFB were reviewed; comparison was performed of the 2 scores in association with exercise capacity and health status. This study showed that as the severity of bronchiectasis increased, walking distance significantly decreased and health status was significantly worsened in both FACED and BSI scoring. Furthermore, there was a significant negative correlation between BSI and walking distance, which was a stronger negative correlation than with FACED. Similarly, there was a significant negative correlation between health status and both FACED and BSI, but this correlation was stronger in the BSI score.

In McDonnell et al.’s European bronchiectasis cohorts, nearly 60% of the patients were female (60% in Scotland, 58% in Italy, 51% in Belgium, and 70% in Serbia) [11]. However, 83% of the patients in our cohort were male. Our hospital is an education and training hospital which serves many districts and generally serves severe patients. Additionally, all of the patients were referred to our pulmonary rehabilitation clinic for consideration and evaluation of a PR program. According to our country’s economic and social status, male patients may have more opportunity to obtain the best health care in the intercity referral system between hospitals. Therefore, this male predominance may be due to the patient referral system in our hospital’s responsive region. 

In this cohort, we have taken radiologically diagnosed NCFB patients with or without airway obstruction. It is quite hard to distinguish pure COPD, bronchiectasis, and overlap syndrome [12,13]. Some questions still remain unanswered in this specific group, as bronchiectasis is frequently diagnosed radiologically in patients with COPD, with different clinical phenotypes. However, for distinguishing these 2 entities, an endotype approach is suggested [14]. According to this approach, a combination of imaging parameters, airway inflammation markers, and microbiology would be used to distinguish between true COPD, bronchiectasis, and the overlap syndrome [14]. In our hospital, we use a combination of imaging parameters, inflammation markers, and sputum cultures for identification of pure bronchiectasis. However, our cohort may have patients overlapping with COPD. In this cohort, the mean FEV1/FVC ratio was 56.8. The majority of the patients had airway obstruction, hence some patients may have overlap syndrome. 

Machado et al. conducted a prospective cohort analysis of 70 patients with NCFB recruited from May 2008 to August 2010 for determining prognostic factors; they found that the mean FEV1% was 48.0 ± 14.8 [15]. In the present study, patients with NCFB had lower FEV1 levels. As we mentioned previously, our hospital is a Step 3 education and research hospital that takes referred severe patients. Therefore, our results may not generalize all NCFB patients in our region. 

In the European cohort, the mean BSI scores ranged from 6 to 9.7 while the mean FACED score was between 1.5 and 2.3 [13]. In the present cohort, the mean FACED score of the population was 3.4 ± 1.5 while the mean BSI score was 8.9 ± 4.6. Our results are quite similar with those of the existing literature. In McDonnell et al.’s study consisting of 7 cohorts, the cohorts were primarily classified as moderate-to-severe bronchiectasis based on mean BSI scores (6.0–9.7); however, in contrast, the majority were classified as mild bronchiectasis according to the FACED score (mean 1.5–2.3). In our study, the majority of the patients were moderate based on the FACED score; however, based on the BSI scores, the majority of the patients were severe. When we add ER visits and hospitalizations to the scoring system, it is seen that there is a tendency for more patients to settle in the severe group. 

These 2 scoring systems were developed for obtaining the risk of mortality; however, these scores also correlate well with exercise capacity. In this present cohort, according to FACED bronchiectasis classification, severe patients had significantly lower 6mWD scores compared to mild and moderate groups. The same was true for the BSI score: according to the BSI index, patients in the mild group had significantly higher 6mWD scores. Thus, we believe that classifying the severity of the patients according to these scoring systems may reflect the exercise capacity of NCFB patients. 

In the same European cohort study, McDonnell et al. suggest that the BSI is superior to FACED in predicting clinically important disease-related outcomes, including hospital admissions, exacerbations, QoL, respiratory symptoms, 6mWD, and lung function decline in bronchiectasis [11]. In this present cohort, the correlation between BSI and SGRQ total score was stronger than for FACED, which is similar to the European results. 

However, the literature shows different results in different etiologies. In a study conducted by Wang et al. to evaluate the clinical characteristics and validation of bronchiectasis severity score systems for posttuberculosis bronchiectasis, the authors found that both FACED and BSI can predict mortality in posttuberculosis bronchiectasis [16]. These data confirm that both scoring systems are excellent predictors of medium-term mortality in subjects with bronchiectasis; however, in a single center retrospective cohort study by Ellis et al., both scores were able to predict 15-year mortality, with the FACED score showing slightly superior predictive power (AUC 0.82 versus 0.69, P = 0.0495) [17]. Although BSI is more closely correlated with health status and exercise capacity in the present study, FACED may be better than BSI in some aspects, among them being the simplicity of its use and its clinical applicability, as shown in the literature [17].

When dealing with bronchiectasis in our daily clinical practice, it is sometimes difficult to decide which score is the best for the individual patient [18]. With our results, we may say that both scoring systems predicted the exercise capacity and health status correctly in NCFB patients. However, if we would like to make a comprehensive and detailed exercise program for NCFB patients, we would prefer to use the BSI score because of its stronger relation with 6mWD and health status scores.

One of the limitations of our study is its retrospective design. However, we have used a detailed data recording system in order to eliminate this limitation. Additionally, NCFB patients referred to our PR unit were symptomatic patients with airflow limitation in need of pulmonary rehabilitation; therefore, our findings may not be generalized to all NCFB patients.

### 4.1. Conclusion

This study showed that as the severity of bronchiectasis increased, walking distance significantly decreased and health status significantly worsened, which was reflected in both FACED and BSI scoring. However, the relationship between 6mWD and health status scores was stronger in the BSI scoring system compared to the FACED bronchiectasis severity score. 

## Statement of ethics

The study was approved by the local institutional review board in January 2017. 
